# Handgrip Strength and Factors Associated in Poor Elderly Assisted at a Primary Care Unit in Rio de Janeiro, Brazil

**DOI:** 10.1371/journal.pone.0166373

**Published:** 2016-11-10

**Authors:** Valéria Teresa Saraiva Lino, Nádia Cristina Pinheiro Rodrigues, Gisele O’Dwyer, Mônica Kramer de Noronha Andrade, Inês Echenique Mattos, Margareth Crisóstomo Portela

**Affiliations:** 1 Department of Primary Care, National Public Health School, Oswaldo Cruz Foundation, Rio de Janeiro, Rio de Janeiro, Brazil; 2 Department of Epidemiology, National Public Health School, Oswaldo Cruz Foundation, Rio de Janeiro, Rio de Janeiro, Brazil; 3 Department of Health Administration and Planning, National Public Health School, Oswaldo Cruz Foundation, Rio de Janeiro, Rio de Janeiro, Brazil; West Virginia University School of Medicine, UNITED STATES

## Abstract

**Introduction:**

Sarcopenia is a condition diagnosed when the patient presents low muscle mass, plus low muscle strength or low physical performance. Muscle weakness in the oldest (dynapenia) is a major public health concern because it predicts future all-cause mortality and is associated with falls, disability, cardiovascular mortality and morbidity. Grip strength is a simple method for assessment of muscle function in clinical practice.

**Objective:**

To estimate the grip strength and identify factors associated with handgrip strength variation in elderly people with low socioeconomic status.

**Methods:**

Cross-sectional study based on a multidimensional assessment of primary care users that were 60 years or older. The sample size was calculated using an estimated prevalence of depression in older adults of 20%. A kappa coefficient of 0.6 with a 95% confidence interval was used to generate a conservative sample size of 180 individuals. *Procedures*: tests and scales to assess humor, cognition (MMSE), basic (ADL) and instrumental activities (IADL) of daily living, mobility (Timed Up and Go), strength, height, Body Mass Index (BMI) and social support were applied. Questions about falls, chronic diseases and self-rated health (SRH) were also included. *Statistical Analysis*: Mean, standard deviation and statistical tests were used to compare grip strength means by demographic and health factors. A multivariate linear model was used to explain the relationship of the predictors with grip strength.

**Results:**

The group was composed predominantly by women (73%) with a very low level of education (mean 3 years of schooling), mean age of 73.09 (± 7.05) years old, good mobility and without IADL impairment. Mean grip strength of male and female were 31.86Kg (SD 5.55) and 21.69Kg (SD 4.48) [p- 0.0001], respectively. Low grip strength was present in 27.7% of women and 39.6% of men. As expected, men and younger participants had higher grip strength than women and older individuals. In the adjusted model, age (p- 0.03), female sex (p- 0.0001), mobility (p- 0.05), height (p- 0.03) and depression (p- 0.03) were independently associated with low grip strength. For every second more in the mobility test, there was a mean decrease of 0.08 Kg in the grip strength. Elders with depression had a mean reduction of 1.74Kg in the grip strength in relation to those in the comparison groups. There was an average reduction of 8.36Kg in the grip strength of elderly females relative to males. For each year of age after 60 years, it was expected an average reduction of 0.11 Kg in the grip strength.

**Conclusion:**

our results suggest that low grip strength is associated with age, female sex, height, depression and mobility problems in poor elderly. Grip strength can be a simple, quick and inexpensive means of stratifying elders’ risk of sarcopenia in the primary care setting. Efforts should be made to recognize weaker persons and the conditions associated to low grip strength in order to target early interventions to prevent frailty and disability.

## Introduction

Sarcopenia is a condition characterized by low muscle mass and function (strength and performance). Skeletal muscle function is an important component of health, ageing, and disease [1] and the mechanisms proposed for the occurrence of sarcopenia in older adults are inflammation, poor nutritional status, and decline in neuromuscular function, hormonal changes and chronic diseases [2,3]. The European Working Group on Sarcopenia in Older People (EWGSOP) has recognized sarcopenia as a syndrome and suggests this diagnosis when the patient presents low muscle mass, plus low muscle strength or low physical performance (measured by gait speed) [4].

Grip strength is the only assessment technique recommended for the measurement of muscle strength and is a simple method for assessment of muscle function in clinical practice [5]. Grip strength declines with increasing age and this kind of muscle weakness in the oldest (dynapenia) is a major public health concern because it predisposes to poorer function and greater risk of many diseases [2]. It predicts future all-cause mortality [1,6], and is associated with falls [7], disability [8] cardiovascular mortality and morbidity [1]. Given its predictive validity and simplicity, grip strength should be considered as a useful measure for the screening of the health conditions of elderly individuals [6] in clinical settings, particularly in the primary care setting, where an appropriate selection of vulnerable older adults could lead to preventive and therapeutic interventions [5].

Although the term dynapenia has been used to define reduced strength, and robust studies stand out that it is a prevalent condition in the older adults, relevant questions remain with regard to its precise definition, including clinically significant cut-off points. Manini and Clark propose that there is still a *work in progress* in the definition of an algorithm specific for dynapenia, arguing that, among other things, there is insufficient data to define clinically relevant cut points for muscle strength [9], and different points lead to different dynapenia prevalence. Alexandre *et al* evidenced the association of dynapenia with mortality in an elderly population of a large Brazilian city using the cut-off suggested by the European group EWGSOP (cut-off values < 30 kg for men and < 20 for women) [10]. Dodds et al defined low grip strength as the strength at least 2.5 SD below the mean gender- specific peak and established normative data for dynapenia combining more than 60 thousand observations [11]. In addition, Fried et al established that weakness was the grip strength in the lowest 20% at baseline, adjusted for gender and body mass index [12].

Literature supports the use of grip strength as an important test for older adults’ examination and even that it should be considered as a vital sign for screening older adults [6]. However, many studies are still required in different countries, in order to reach a consensus on dynapenia and how it should be assessed before the routine use by clinicians. The present study estimated the grip strength and identified factors associated with its variation in elderly people with low socioeconomic status assisted at a primary care unit and subject to great environmental vulnerability in a large city in Brazil.

## Materials and Methods

### Study Setting and Sample

This study was part of an investigation aimed at developing a screening strategy for elderly patients based on a comprehensive geriatric assessment, performed at the primary care unit of the Oswaldo Cruz Foundation, in the city of Rio de Janeiro, Brazil. This unit provides services to 31,000 residents of the Manguinhos District, where most houses have a single room, sanitary issues are still unsolved, monthly family income is usually lower than a the minimum wage, and more than 50% of residents have no more than an elementary school education [13]. Initiatives aimed at decreasing violence have been impeded by the activities of local drug traffickers [14].

The convenience sample was drawn from users that were 60 years or older who received outpatient care from the Family Health Team, selected for their accessibility to the primary care unit. Health professionals, researchers or other elderly individuals invited them to participate in the research. Those with advanced cognitive and sensorial deficits or impaired locomotion were excluded. The sample size was calculated using the expected proportion of 20% of two common health problems in elderly individuals, such as dementia and depression. [15]. A kappa coefficient of 0.6 with a 95% confidence interval was used to generate a conservative sample size of 180 individuals, estimated using the Win- Pepi application, version 2009 [16].

### Procedures

Data collection occurred from June to December 2010. In order to perform this study, the following tests and scales were used: Independence in Activities of Daily Living Index [17,18], Functional Activities Questionnaire [19], Timed Up and Go (the examinee gets up from a chair and walks a distance of 3m quickly. If more than 10 seconds is necessary, there is a mobility disorder) [20], Structured Clinical Interview for DSM-IV—clinical version [21], Mini-Mental State Examination) [22,23], Body Mass Index (BMI) [24] and the Medical Outcomes Study Social Support Scale [25,26]. Questions about falls (two or more falls during the last year) and self-rated health (SRH) [27] were also included. The assessment of grip strength was performed with the Dynamometer Crown^®^ and the volunteer sitting with the forearm in the pronated position, the elbow in 90° flexion and shoulders adducted. After positioning the volunteer, he/she was encouraged to squeeze the dynamometer as hard as he/she could. The dominant hand was assessed twice, alternating with the non-dominant hand and the stronger grip was registered as the individual grip strength. Normal and altered grip strength values were established according to criteria proposed by Fried *et al* [12].

After performing the geriatric assessment, patient’s records were examined to verify the diseases reported.

### Statistical Analysis

Descriptive analysis of the study population characteristics were performed. Mean, standard deviation and statistical tests were used to compare grip strength means by sex, age group (< 73; ≥ 73 years old), height (meters), BMI, education (< 3; ≥ 3 years of schooling), SRH, asthma, hypertension, diabetes, arthritis, Chronic Obstructive Pulmonary Disease (COPD), comorbidities (considered as ≥ 3 chronic diseases asthma, Hypertension, COPD, Diabetes and Arthritis), depression, mobility, falls, cognition, IADL and social support.

A multivariate linear model was used to explain the relationship of the predictors with grip strength (response variable in Kg). The factors included in the first model were self-rated health, depression, comorbidities, mobility, height, sex, age and cognitive dysfunction.

Model:

$$Y\sim N\left(\mu ,\sigma^{2}\right)$$

$$E\left(grip\;strength\right)=\alpha +\beta_{1}*depression+\beta_{2}*self-rated\;health+\beta_{3}*mobility+\beta_{4}*age+\beta_{5}*sex+\beta_{6}*height+\beta_{7}*cognitive\;dysfunction+\beta \_8*comorbidities$$

where, *μ* is the mean, σ^2^ is the variance, E is the expected value, α is the model intercept and each β represents the regression coefficients of independent variables. All analyses were performed with R-Project (version 3.2.4) software.

### Ethics

The Ethics Research Committee of the National Public Health School/Fiocruz (report number 126/10) approved the research. All participants signed an informed consent form, which pledges anonymity and confidentiality of the information.

## Results

The sample was composed predominantly by women with a very low level of education, good mobility and without IADL impairment. Handgrip strength presented a mean (std) of 21.69 (4.48), among women, and a mean of 31.86 (5.55), among men (Table 1). Overall, 30.9% of the elderly had dynapenia, being the prevalence of the problem among women (27.3%) lower than that observed among men (39.6%).

**Table 1:** Characteristics of the participants in the study according to demographics and health status.

Parameters	Estimate (N = 180)
Age—mean (SD)	73.09 (7.05)
Gender (women)—n (%)	132 (73.00)
Education (years of schooling): mean (SD)	3.01 (2.93)
Mobility—TUG (seconds): mean (SD)	12.55 (9.27)
Height—mean (SD)	1.54 (0.08)
Women—mean (SD)	1.50 (0.06)
Men—mean (SD)	1.62 (0.06)
BMI (Kg/m^2^)—mean (SD)	28.70 (5.40)
Women—mean (SD)	29.21 (5.95)
Men—mean (SD)	27.55 (3.46)
Grip strength—Kg: mean (SD)	24.42 (6.57)
Women—mean (SD)	21.69 (4.48)
Men—mean (SD)	31.86 (5.55)
Low grip strength—n (%)	55 (30.89)
Women—n (%)	36 (27.27)
Men—n (%)	19 (39.60)
IADL (dependent)—n (%)	50 (28.00)
Falls—n (%)	42 (23.00)
Depression—n (%)	52 (29.00)
Unsatisfactory social support—n (%)	46 (25.60)
Self-rated health—n (%)	
Good	100 (56.00)
Fair	60 (33.00)
Poor	20 (11.00)
Cognitive dysfunction—n (%)	81 (45.25)
Arterial hypertension: n (%)	151 (83.90)
Arthritis—n (%)	77 (42.78)
Diabetes—n (%)	56 (31.10)
COPD—n (%)	21 (11.70)
Asthma—n (%)	13 (7.20)
Comorbidities (≥3)—n (%)	35 (19.40)

BMI-Body Mass Index; COPD- Chronic Obstructive Pulmonary Disease; IADL- Instrumental activities of daily living; SD- standard deviation; TUG- Timed Up and Go.

As expected, men, younger participants and the ones with greater height had higher grip strength than women, older and lower individuals. The analysis of the relation of the height and grip strength stratified by sex demonstrated association of grip strength and height only in women (p-value: 0.005).

Those with either better mobility or good SRH were stronger than those in the comparison categories. Arthritis, cognitive dysfunction and depression were the conditions with the worse grip strength (Table 2), being worth noting that in the patients’ records, there were only three individuals with psychiatric disorders diagnosis. In the adjusted model, there was no association between low grip strength and chronic diseases like diabetes, COPD, hypertension and asthma. BMI showed no association either. Cognitive dysfunction, comorbidities, SRH and arthritis were associated with grip strength, but after adjustment, the association disappeared. Age, sex, height, mobility and depression were independently associated with decreased muscle strength. The greater the height, the greater the grip strength. For every second more in the mobility test, there was a mean decrease of 0.08 Kg in the grip strength. So, there was a mean reduction of 2.16 Kg in the grip strength of the elderly with reduced mobility compared to those without mobility alterations. Depressed elderly had a mean reduction of 1.74 Kg in the grip strength in relation to the not depressed ones. A borderline statistical result (p-value < 0.09) suggests a reduction in the grip strength of elders with poor SRH in relation to those with good SRH. There was an average reduction of 8.36 Kg in the grip strength of elderly females relative to males. For each year of age after 60 years is expected an average reduction of 0.11 Kg in the grip strength. (Table 3).

**Table 2:** Relation of the mean grip strength (Kg) with demographic and health parameters.

Parameters	n	Categories	Grip strength	p-value
			Mean (SD)	
Sex	48	Male	31.86 (5.55)	0.0001
	132	Female	21.69 (4.48)	
Age group (years)	113	< 75	25.11 (6.65)	0.06
	67	≥ 75	23.24 (6.32)	
Height	45	≤ 1.48	20.11 (3.63)	0.0001
	46	1.48–1.53	22.01 (5.31)	
	43	1.53–1.58	25.89 (6.00)	
	43	> 1.58	29.98 (6.62)	
BMI	23	Low weight/Eutrophic	23.09 (7.27)	0.35
	157	Overweight/Obesity	24.62 (6.47)	
Study years	102	< 3	24.50 (6.80)	0.86
	78	≥ 3	24.32 (6.31)	
Self-rated health	160	Good/Very good	24.90 (6.55)	0.004
	20	Bad/Very bad/Moderate	20.63 (5.55)	
Asthma	167	No	24.57 (6.54)	0.33
	13	Yes	22.54 (7.05)	
Hypertension	31	No	25.48 (7.11)	0.36
	150	Yes	24.20 (6.49)	
COPD	159	No	24.24 (6.32)	0.44
	21	Yes	25.74 (8.32)	
Diabetes	123	No	24.44 (6.64)	0.95
	57	Yes	24.38 (6.48)	
Arthritis	103	No	25.95 (7.20)	0.0001
	77	Yes	22.35 (4.94)	
Comorbidities^1^	35	< 3	24.94 (6.58)	0.02
	145	≥ 3	22.19 (6.15)	
Depression^2^	128	No	25.39 (6.57)	0.002
	53	Yes	22.16 (6.02)	
Mobility (seconds)	71	< 10	26.41 (6.69)	0.001
	111	≥ 10	23.15 (6.24)	
Falls	138	No	24.39 (6.90)	0.91
	42	Yes	24.51 (5.47)	
Cognitive^3^ dysfunction	99	No	25.49 (6.87)	0.02
	82	Yes	23.14 (6.02)	
IADL^4^	130	Independent	24.78 (6.40)	0.25
	50	Dependent	23.49 (6.99)	
Poor social support^5^	134	No	24.74 (6.56)	0.26
	46	Yes	23.48 (6.59)	

^1^numbers of chronic diseases;

^2^Acessed by DSM-IV (Diagnostic and Statistical Manual of Mental Disorders- American Psychiatry Association);

^3^Accessed by Mini-Mental State Exam score combined to the number of study years;

^4^Accessed by Functional Activities Questionnaire;

^5^Accessed by Medical Outcomes Study score.

BMI = Body Mass Index; COPD = Chronic Obstructive Pulmonary Disease; IADL = Instrumental Activities of Daily Living; SD = Standard Deviation.

**Table 3:** Adjusted relation of grip strength with demographic and health parameters.

Parameters	Coeff	CI 95%	p-value
Mobility (seconds)	-0.08	-0.16; -0.01	0.05
Depression	-1.74	-3.31; -0.18	0.03
Self-rated health (reference: Good/Very good)	-1.95	-4.19; 0.37	0.10
Sex (reference: male)	-8.36	-10.38; -6.18	0.0001
Age (years)	-0.11	-0.21; -0.01	0.03
Height (meters)	14.00	1.54; 24.46	0.03
Cognitive dysfunction	-0.74	-1.98; 0.75	0.37
Comorbidities	-0.89	-2.65; 0.88	0.32

CI = Confidence Interval; Coeff = Adjusted Regression Coefficient; Coefficients were calculated using two linear models to access the relationship between grip strength and the explanatory variables.

## Discussion

In this research, low grip strength was a common condition in poor elderly with a prevalence of 27.7% in women and 39.6% in men. Dodds *et al*, identified low muscle strength in 23% of males and 27% of females by age 80 in more than 60 thousand observations from 49,964 British individuals [11]. Alexandre *et al* also demonstrated that dynapenia can be common, with prevalences of 29.5% and 41% in two Brazilian samples of 478 and 1149 elders living in the community, respectively [10,28].

As expected, an inverse association between strength measures and age was observed from 60 years onwards in our study. For each year of age after 60 years it was expected an average reduction of 0.11 Kg in the grip strength, a decrease of almost 1 Kg for each decade of life. This is not a surprisingly finding as the decline in grip strength begins at the fifth decade of life in both men and women [11]. Examining almost 140 thousand individuals in a longitudinal study involving 17 countries of varying incomes and sociocultural settings, Leong *et al* verified that high grip strength was also associated with young age [29].

We found no association between education and grip strength. Educational attainment is a well-established social determinant of health and it could be expected an association of education with lower grip strength, a predictor of frailty and disability in old age. However, the effects of education are inconsistent and usually disappear after adjustments for health measures [30–32].

The association between weakness and chronic diseases have been reported. In their robust longitudinal study, Leong et al found association between low grip strength and baseline presence of hypertension, coronary artery disease, stroke and COPD, but not diabetes [29]. Stenholm et al examined long-term changes in muscle strength and verified that diabetes, hypertension, asthma, obesity and the presence of two or more chronic diseases were predictors of decline in muscle strength over a follow-up period of 22 years in average, in a population with age varying from 30 to 73 years at baseline [2]. These conditions can explain potential mechanisms of dysregulation of physiological systems and muscle strength decline. Obesity have catabolic effects on muscle through inflammation and insulin resistance leading to sarcopenic obesity. The same can be said about diabetes. Vascular diseases can produce vascular-related damage to the musculoskeletal and peripheral nervous systems [2]. Our results neither demonstrated association of decreased grip strength with chronic diseases individually (asthma, COPD, hypertension, obesity and diabetes) nor did it with comorbidities or cognitive dysfunction. Maybe the sample size and the reduced number of individuals with low grip strength (55) can explain this occurrence.

As the muscle mass and function of men is higher than that of women, another expected finding was the lower grip strength in women. There was a 10Kg difference between genders. This is the same difference reported in the definition of dynapenia which requires grip strength lower than 30Kg and 20Kg for men and women, respectively [4]. In agreement with the present study, Leong *et al* also noted such difference between genders in low, medium and high-income countries. In middle-income ones, including Brazil the mean grip strength in men was 37.3Kg (SD 8.19) and 27.9Kg (SD 8.07) in women [29].

As demonstrated by other authors [29,33], we also verified association between grip strength and height, meaning that taller individuals had better grip strength. When it was stratified by sex, the association persisted only in women (p-value: 0.005) but this can be attributed to the small number of men in the sample.

We verified that dynapenia was associated with decreased performance in the mobility test, a natural occurrence as the muscle strength measures of different body compartments are correlated. Grip strength can be a reliable surrogate for measures of muscle strength in the legs [4]. In Brazil, Bez & Neri examined 689 elders to analyze the association between SRH and gait speed/ grip strength and also found a correlation between dynapenia and slow gait speed. The octogenarians were the slowest and weaker group [34].

SRH is a subjective measure based on values and criteria related to individual and social expectations as well as social and temporal comparison mechanisms [34]. It has been applied as an outcome measure for overall or ‘true’ health [35] and it is associated with disability in the elderly people. Our results demonstrated a borderline association between SRH and dynapenia. Good SRH was positively associated with higher levels of muscle strength in other studies [33,35] and the associations seem biologically plausible.

In this study depression was unequivocally associated with low grip strength, demonstrating a biological plausibility linking sarcopenia and depression. Other studies report association of sarcopenia with depression. Frailty, identified through Fried’s biological model, was associated with a higher likelihood of depression and anxiety in community dwelling elderly in Ireland (OR 4.3, 95% CI 1.5–11.9) [36]. In a cross-sectional Korean study, also involving men and women aged 60 years and older, individuals with self-reported depression or those taking antidepressants had lower appendicular skeletal muscle mass than those free of depression [37]. And in a prospective cohort study with a 1-year follow-up conducted with community-dwelling individuals in Japan, subjects with lower grip strength had higher odds of having depressive symptoms at baseline and after 1 year [adjusted odds ratio 1.15, 95% confidence interval (CI) 1.06–1.24; and 1.13, 95% CI 1.01–1.27, respectively] [38].

Those studies demonstrate that sarcopenia and depression possibly share a common pathophysiological pathway. The skeletal muscle production of brain-derived neurotrophic factor (BDNF), inflammation and oxidative stress play a role in the modulation of mood and anxiety. BDNF participates in the neuronal differentiation and survival, but in the skeletal muscle it is involved in the maintenance of motor units and reduces the denervation-induced atrophy seen in sarcopenia. Physical exercise enhances circulating BDNF, down-regulates systemic inflammation and is recognized as an effective treatment for depression and anxiety. Antidepressant treatment normalizes markers of inflammation and oxidative stress [39].

The instrument used in this study deserves a comment. We presume that it has produced acceptable measurements because it is known that the use of different dynamometers doesn’t seem to affect the strength measures, producing good similar data [6,11]. As we had already checked the reliability of grip strength with our tool [40], we can conclude that it also provided suitable results.

The criterion used to establish weakness in this work also deserves a comment. Fried *et al* established that weakness was the grip strength in the lowest 20% at baseline, adjusted for gender and body mass index, identifying its association with frailty [12]. The same criterion has been used in other studies, including a study in Brazil, indicating clinically relevant endpoints [41]. We identified the association of dynapenia in a poor population of a big city, subject to great environmental vulnerability with health outcomes using Fried’s criterion. This suggests the clinical relevance of Fried’s cut-off point in such an environment.

Our study presents limitations, but also strengths. Depression and sarcopenia potentially share a common pathophysiological pathway. As the study design had not considered data collection for depression treatment, the association between grip strength and depression could be biased by the omission of that variable. We believe, however, that the overwhelming majority of volunteers diagnosed as depressive in our study was not under treatment, given that only three medical records had psychiatric diseases registered. Physical activity performed by the volunteers was either not collected, and we recognize that its effect on depression and anxiety unaccounted could also potentially interfere on the association between depression and grip strength. Other aspects related to the lifestyle which are associated with grip strength, like dietary factors were not investigated. Since data is cross-sectional, the study does not allow for causality considerations. We used a convenience sample of not impaired older adults from a primary care setting in a low-income area and this choice can lead to a risk of selection bias. Also, the results are not generalizable. However, the population represented is similar to those who reside in outlying suburbs of Brazilian metropolitan areas. Also, we performed direct examination of individuals in most features, instead of considering the self-report of health variables, which must be interpreted with caution [33]. And, we eventually used tools that were validated in Brazil.

## Conclusion

Our results suggest that low grip strength is associated with older age, female sex, depression, reduced height and dismobility. Grip strength can be a simple, quick and inexpensive means of stratifying elders’ risk of sarcopenia in the primary care setting. Efforts should be made to recognize weaker persons and the conditions associated to low grip strength in order to target early interventions to prevent frailty and disability.
